# Association of Phthalate Exposure with Respiratory and Allergic Symptoms and Type 2 and Non-Type 2 Inflammation: The Hokkaido Study

**DOI:** 10.1021/acs.est.4c14579

**Published:** 2025-04-08

**Authors:** Rahel Mesfin Ketema, Yu Ait Bamai, Houman Goudarzi, Takeshi Yamaguchi, Yi Zeng, Ayaka Yasuda, Megasari Marsela, Satoshi Konno, Reiko Kishi, Atsuko Ikeda

**Affiliations:** † Faculty of Health Sciences, Hokkaido University, Kita 12 Nishi 5, Kita-ku, Sapporo 060-0812, Japan; ‡ Center for Environmental and Health Sciences, Hokkaido University, Kita 12, Nishi 7, Kita-ku, Sapporo 060-0812, Japan; § Faculty of Medicine, Hokkaido University, Kita 15, Nishi 7, Kita-ku, 060-8638 Sapporo Japan; ∥ Creative Research Institution, Hokkaido University, North 21, West 10, Kita-ku, Sapporo 001-0021, Japan; ⊥ Graduate School of Pharmaceutical Sciences, Health Sciences University of Hokkaido, 1757 Kanazawa, Tobetsu-cho, Ishikari-gun 061-0293, Japan; # Graduate School of Health Sciences, Hokkaido University, Kita 12 Nishi 5, Kita-ku, Sapporo 060-0812, Japan

**Keywords:** phthalates, children, type 2 (T2) inflammation, non-T2 inflammation, mixture effect

## Abstract

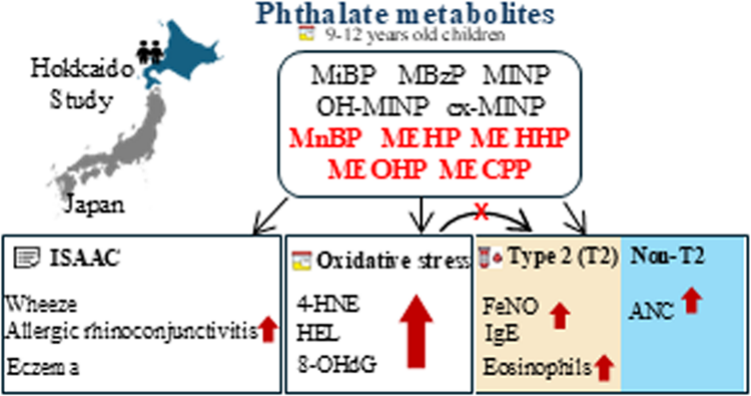

Phthalate exposure is linked to asthma and allergic symptoms, yet their individual and combined effects on symptoms and inflammatory biomarkers, type 2 (T2) and non-T2, remain unexplored. This study examined the association of phthalate metabolites with allergic symptoms (wheeze, allergic rhinoconjunctivitis, and eczema), T2 biomarker (fraction of exhaled nitric oxide (FeNO), blood eosinophil count, and total immunoglobulin E (IgE)), and non-T2 biomarker (absolute neutrophil count (ANC)) and also their association with oxidative stress biomarkers, such as 4-hydroxynonenal, hexanoyl-lysine, and 8-hydroxy-2-deoxyguanosine. Ten urinary phthalate metabolites were measured using UPLC-MS/MS in 421 children (aged 9–12 years) from The Hokkaido Cohort, Japan. Symptoms were defined using the International Study of Asthma and Allergies in Childhood questionnaire, and biomarkers were measured in blood. Logistic regression assessed individual metabolites, while quantile-g computation and Bayesian kernel machine regression analyzed mixture effects on binary outcomes. Individual analysis showed that MnBP (mono-*n*-butyl phthalate) was positively associated with allergic rhinoconjunctivitis and eosinophil ≥ 300 cells/μL, while ∑DBP (dibutyl phthalate) and OH-MiNP (mono-hydroxy-isononyl phthalate) were linked with FeNO ≥ 35 ppb. DEHP (di(2-ethylhexyl) phthalate) metabolites were associated with a high prevalence of blood eosinophils ≥ 300 cells/μL. We found a positive association between phthalates and oxidative stress markers, but no link was observed between oxidative stress and inflammatory markers. Mixture analysis identified MnBP as a major contributor to the high FeNO level, with di-*n*-butyl phthalate (DnBP) and DEHP metabolites contributing to eosinophil count ≥ 300 cells/μL and ANC ≥ 4400 cells/μL. These findings suggest that phthalate exposure from DnBP and DEHP is associated with immune dysregulation by triggering both T2 and non-T2 inflammatory responses.

## Abstract 2

Phthalate exposure, notably DBP and DEHP, promote inflammatory biomarkers linked to asthma and allergies, underscoring the importance of stricter and improved regulation to protect public health.

## 1 Introduction

Phthalates are chemicals extensively used in various manufacturing and consumer products as plasticizers or solvents.^1^ This leads to their ubiquitous environmental presence. Human exposure to phthalates occurs primarily through ingestion, inhalation, and dermal contact, leading to widespread concerns about their potential health impacts.^2^

Among their various health effects, phthalates have been a concern for exacerbating asthma and allergic symptoms.^3^ Epidemiological studies have reported that phthalate exposure may contribute to the development of asthma and allergic conditions of wheeze, allergic rhinoconjunctivitis, and eczema.^4−9^ For instance, the Swedish SELMA study revealed that prenatal exposure of butyl benzyl phthalate (BBzP) and diisononyl phthalate (DINP) was positively associated with wheeze in children.^8^ Our previous research in Hokkaido, Japan, has also explored the effect of phthalates on children’s health. We identified a link between floor dust di(2-ethylhexyl) phthalate (DEHP) and increased allergic rhinoconjunctivitis in children.^9^ Additionally, our investigation in urine showed significant positive association of DEHP and DINP metabolites with wheeze and eczema in 7-year-old children.^4^ Systematic reviews further support these findings, indicating that both prenatal and early life exposure to phthalates increase the risk of asthma and allergies in children.^10,11^

While these studies link phthalate exposure with increased asthma and allergic symptoms, they often overlooked its impact on type 2 (T2) inflammatory biomarkers, such as fractional exhaled nitric oxide (FeNO), immunoglobulin (IgE), and eosinophils, and the non-T2 biomarker neutrophils. Previous studies suggest that phthalate exposure may alter T2 and non-T2 biomarkers.^6,12−15^ For instance, one study reported that urinary levels of BBzP were linked to elevated FeNO levels, with this association being more prevalent in children with wheeze symptoms.^12^ Additionally, a study on school children found that urinary phthalate metabolite levels were significantly associated with increased blood eosinophil count.^14^ Interestingly, our previous study found that a higher amount of maternal DEHP metabolite MEHP is negatively associated with cord blood IgE and is linked to increased risk of allergies and infectious diseases in children up to age seven.^6^ In contrast, a Taiwan birth cohort that examined prenatal phthalates reported a positive association with serum IgE in 2 year old boys.^15^ Regarding non-T2 biomarkers, National Health and Nutrition Examination Survey (NHANES) data between 1999 and 2006 revealed a positive relation with absolute neutrophil count (ANC), especially with DEHP and DBP metabolites.^13^ The majority of childhood asthmatic patients exhibit T2 inflammation, along with non-T2 inflammation occurring to a lesser extent in some cases.^16^ It is worth mentioning that non-T2 inflammation is characterized by few biomarkers and sometimes leads to poor prognosis.^17^ Therefore, it is important to examine both T2 and non-T2 inflammation to achieve a comprehensive understanding of pathogenesis in asthma. Furthermore, the studies mentioned above report inconsistent results, and to date no study has examined both T2 and non-T2 inflammatory biomarkers simultaneously to investigate their association with phthalate exposure. Addressing this gap is essential to understanding how phthalate exposure influences distinct inflammatory pathways and contributes to respiratory and allergic conditions. In addition to inflammation, oxidative stress is another important mechanism through which phthalates may contribute to the development of asthma and allergies. Studies suggest that phthalates can increase oxidative stress biomarkers such as 8-hydroxy-2-deoxyguanosine (8-OHdG),^18^ while others report no association.^4^ An in vivo study suggests the potential mediating role of phthalates in contributing to asthma and allergic symptoms.^19^

Additionally, previous studies often examine individual chemicals while potentially overlooking the simultaneous exposure of humans to multiple chemicals and their mixture effect. To address this issue, mixture analyses such as quantile-g computation (qgcomp) and Bayesian kernel machine regression (BKMR) have been introduced.^20,21^

Thus, this study aims to investigate the association of individual and mixture effects of phthalate metabolites with allergic symptoms (wheeze, allergic rhinoconjunctivitis, and eczema), T2 biomarkers (FeNO level, IgE, and eosinophil count), and non-T2 biomarkers (blood neutrophil count). An additional aim of this study is to examine the association of phthalates and health outcomes with oxidative stress biomarkers, such as 4-hydroxynonenal (4-HNE), hexanoyl-lysine (HEL), and 8-OHdG. The study participants are healthy children and are not limited to allergic cases. This approach broadens generalizability, offering insights into inflammatory pathways in asthma and allergies while identifying key phthalates and providing valuable public health implications.

## 2 Materials and Methods

### 2.1 Study Population

This study is part of the ongoing Hokkaido cohort “Hokkaido Study on Environment and Children’s Health”.^22−25^ Details of participant selection are described elsewhere.^26^ Briefly, we contacted all eligible children who reached the age of 9–12 years between September 2017 and March 2020 in Sapporo city, Japan. To avoid selection bias, we intentionally refrained from stating allergic diseases as an outcome in the invitation letters.^27^ We received 428 participants who agreed to a face-to-face survey and visited pediatric clinics with their parents.^26,27^ During the pediatric visit, physical examinations were conducted and urine and blood samples were collected. Additionally, parents were asked to answer the International Study of Asthma and Allergies in Childhood (ISAAC) questionnaire. Seven children were excluded from the analysis due to insufficient urine volume and errors during the sample pretreatment procedure. A total of 421 children, with completed questionnaires, urine samples, and blood sample data, were included in the current study.

### 2.2 Questionnaire

All questionnaires were completed by the parents of the children. The survey included demographic characteristics, such as sex, age, height, weight, annual household income, environmental tobacco smoke (ETS) (Yes/No), pet in the house (Yes/No), floor materials (polyvinyl chloride (PVC), Tatami, flooring/tile, and carpet). Allergic symptoms, wheeze, allergic rhinoconjunctivitis, and eczema were assessed using the ISAAC questionnaire.^28^ Wheeze was defined based on a question “Has your child had wheezing or whistling in the chest in the past 12 months?” Current allergic rhinoconjunctivitis symptoms were defined by the “Yes” response to both questions: (a) “Has your child experienced sneezing or a runny/stuffy nose in the absence of a cold or flu in the last 12 months?” and (b) “This child’s nose problem was accompanied by itchy or watery eyes?”. Eczema was defined based on the ‘Yes’ response to all three questions: (a) “Has your child had this itchy rash at any time in the past 12 months?” (b) “Has your child ever had a recurrent skin rash for at least six months?” and (c) “Has this itchy rash at any time affected any of the following places: folds of the elbows, behind the knees, in front of the ankles, under the buttocks, or around the neck, ears, or eyes?”

### 2.3 Measurement of Phthalate Metabolites

We measured 10 urinary phthalate metabolites: monoisobutyl phthalate (MiBP), mono-*n*-butyl phthalate (MnBP), monobenzyl phthalate (MBzP), mono(2-ethylhexyl) phthalate (MEHP), mono(2-ethyl-5-hydroxyhexyl) phthalate (MEHHP), mono(2-ethyl-5-oxohexyl) phthalate (MEOHP), mono(2-ethyl-5-carboxypentyl) phthalate (MECPP), monoisononyl phthalate (MINP), monohydroxy-isononyl phthalate (OH-MINP), and monocarboxy-isononyl phthalate (carboxy-isononyl phthalate) (cx-MINP) in children urine. Details of analytical measurement of metabolites, quality assurance, and quality control have been described elsewhere.^29^ Briefly, an analytical method using deconjugation and solid-phase extraction followed by ultraperformance liquid chromatography/tandem mass spectrometry (UPLC-MS/MS) was employed to measure the phthalate metabolites. For quality assurance, two procedural blanks were analyzed per batch. Calibration curves in all batches showed high accuracy (correlation coefficient, ≥0.998). In each batch of 20 samples, a quality control calibration standard at a concentration of 5 ng/mL and a reference 63 German external quality assessment scheme sample with a known concentration were used to assess the precision, with inter- and intraday variations under a coefficient of variation of 10%. Limits of detection (LODs) ranged from 0.05 to 0.95 ng/mL.

### 2.4 Measurement of T2 Biomarkers

Details of sample collection and analytical methods were reported in our previous studies.^26,27^ Briefly, FeNO levels were measured at the pediatric clinic with a NIOX VERO electrochemical sensor (Aerocrine, Stockholm, Sweden), expressed in parts per billion (ppb). Peripheral blood samples collected from children were analyzed for eosinophil and neutrophil counts at Daiichi Kishimoto Clinical Laboratories, Inc. (Sapporo, Japan). Total IgE levels (IU/mL) were measured in the serum samples at SRL Inc., (Tokyo, Japan) using the enzyme-linked immunosorbent assay (ELISA). Children were categorized based on specific cutoff values for T2 and non-T2 biomarkers. Children with FeNO ≥ 35 ppb were classified according to the American Thoracic Society.^30^ While precise cutoff values for IgE and eosinophils in children are not standardized, eosinophil count ≥ 300 cells/μL and ANC ≥ 4400 cells/μL are commonly referenced.^31^ For the IgE level, a cutoff value of 170 IU/mL was applied, consistent with the Japanese commercial medical test companies and national cohort studies conducted in Japan.^32^

### 2.5 Measurement of Oxidative Stress Biomarkers

Detailed measurements of three oxidative stress biomarkers: 4-HNE, HEL, and 8-OHdG in urine samples have been included in a previous publication.^33^ 4-HNE and HEL were measured at MACROPHI (Kagawa, Japan) using a HEL ELISA kit (Japan Institute for the Control of Aging, Nikken SEIL Co., Shizuoka, Japan) and an OxiSelect HNE Adduct Competitive ELISA kit (Cell Biolabs, San Diego, CA), respectively. 8-OHdG was quantified using an 8-OHdG Check ELISA kit (Japan Institute for the Control of Aging, Nikken SEIL Co., Shizuoka, Japan).

### 2.6 Statistical Analysis

Categorical data are expressed as counts and percentile, while continuous data are expressed as means with standard deviation (SD). Phthalate metabolite concentrations <LOD were imputed with LOD × detection frequency.^34^ The summation of phthalates was conducted with their corresponding metabolites’ molar sum, denoted as ∑DBP (dibutyl phthalate) for MiBP and MnBP, ∑DEHP for MEHP, MEOHP, MEHHP, and MECPP, and ∑DINP for MINP, OH-MINP, and cx-MINP. Metabolite concentrations were corrected for creatinine and were natural logarithm-transformed to enhance the normal distribution for analysis. To select potential confounders for adjustment, we first included all significant variables from Supporting Information Table 1 and literature-based variables for each outcome to examine if their adjustment resulted in a change of odds ratio (OR) > 10%.^35^ Consequently, sex, age, BMI, ETS, and season were selected as confounders and included in each model. The association between individual phthalate metabolites and binomial outcomes of wheeze, allergic rhinoconjunctivitis, eczema, and T2 and non-T2 biomarkers were examined using multiple logistic regression analysis. The odds ratio (OR) and 95% confidence interval (CI) were calculated by using JMP Pro version 16.1.0 (SAS Institute, Inc., Cary, NC). The combined effect of the phthalate metabolite mixture was examined by quantile-g computation (qgcomp) and BKMR. The quantile-g computation, as previously described in detail in Keil et al., estimated the combined effect on the outcome associated with a simultaneous one-quantile increase in the mixture exposure level.^20^ Qgcomp also evaluated combined and individual contributions by computing both positive and negative weights.^20^ For logistic regression and qgcomp, *p*-values less than 0.05 were considered statistically significant. We used the BKMR model, a nonparametric method to assess the mixture effect and individual contribution of phthalate metabolites to outcomes.^21^ We grouped metabolites of the same parent phthalate compound into four groups (group 1: MiBP and MnBP; group 2: MBzP; group 3: MEHP, MEOHP, MEHHP, and MECPP; and group 4: MINP, OH-MINP, and cx-MINP). We used a hierarchical variable selection method with 10,000 iterations to estimate the posterior inclusion probability (PIP) of highly correlated variables. The group probability inclusion (groupPIP) and conditional PIP (condPIP) indicated the probability of the group and the individual metabolite contribution to the outcome. For the overall metabolite mixture effect on outcomes, we compared all metabolites at their 50th percentile as reference. The BKMR overall mixture effect was considered statistically significant if its 95% credible interval did not overlap with zero. The qgcomp (version 2.15.2) and BKMR (version 0.2.0) were conducted with R (version 3.5.1).

#### 2.6.1 Ethics

Approval for this study was obtained from the Ethics Board of the Hokkaido University Center for Environmental and Health Sciences for Epidemiological Studies (21–136). The research objectives and methods were explained to the children and their parents; then, written informed consent was obtained from parents, and permission was obtained from the children involved in this study.

## 3 Results

### 3.1 Participant Characteristics

The general demographic characteristics of the participants are shown in Table 1. A total of 421 children aged between 9 and 12 years, of which 53.9% were male and 46.1% were female were included in this study. Among participants, 31 (7.4%) reported wheeze, 88 (21%) reported allergic rhinoconjunctivitis, and 96 (22.8%) reported eczema. Regarding T2 biomarkers, 113 (26.8%) had FeNO levels ≥ 35 ppb, 181 (42.9%) had serum total IgE levels ≥ 170 (IU/mL), 159 (37.7%) had blood eosinophil levels ≥ 300 cells/μL, and 68 (16.2%) showed non-T2 biomarker levels of ANC ≥ 4400 cells/μL.

**Table 1:** Characteristics of Participants^a^

variable		counts (%)
sex	male	227 (53.9)
	female	194 (46.1)
child age	9	139 (33.0)
	10	168 (39.9)
	11	44 (10.4)
	12	70 (16.6)
BMI (kg/m^2^)	mean (±SD)	17.7 (2.9)
annual household income (million yen)	<3	25 (6.2)
	3–<5	101 (25.3)
	5–<8	176 (44.2)
	≥8	96 (24.1)
ETS	yes	169 (40.1)
pet in the house	yes	112 (26.6)
PVC flooring	yes	38 (9.0)
tatami flooring	yes	22 (5.2)
flooring	yes	293 (69.6)
carpet	yes	48 (11.4)
ISAAC Symptoms		
wheeze	yes	31 (7.4)
allergic rhinoconjunctivitis	yes	89 (21.2)
eczema	yes	95 (22.8)
Biomarkers		
FeNO ≥ 35 ppb	yes	113 (26.5)
total IgE ≥ 170 (IU/mL)	yes	181 (42.9)
eosinophil ≥ 300 cells/μL	yes	159 (37.7)
ANC ≥ 4400 cells/μL	yes	68 (16.2)

a Abbreviations: BMI: body mass index; ETS: environmental tobacco smoke; ISAAC: International Study of Asthma and Allergies in Childhood; FeNO: fraction of exhaled nitric oxide; IgE: immunoglobulin E; ANC: absolute neutrophil count; Counts represent the number of participants in each category.

### 3.2 Distribution of Phthalate Metabolites and Oxidative Stress Biomarkers

The phthalate metabolite distribution in children’s urine is shown in Table 2. All 10 examined phthalate metabolites were present in more than 87% of the children’s urine. Considering the median concentration level, MnBP was the highest at 19.3 ng/mL, followed by MECPP and MEHHP with 13.4 and 10.5 ng/mL, respectively. Significant Spearman correlation was observed among all metabolites, particularly among DEHP metabolites (MEHP, MEOHP, MEHHP, and MECPP) having correlation coefficients greater than 0.676. The distribution of oxidative stress biomarker levels is shown in Table S2. The median levels for HNE, HEL, and 8-OHdG were 25.1 μg/mL, 100.5 nmol/L, and 90.0 ng/mL, respectively.

**Table 2:** Distribution of Urinary Phthalate Metabolite Concentrations in 9–12 Year Old Children^a^

parent compounds	metabolites	*N*	LOD (ng/mL)	>LOD%	min	25%	50%	75%	95%	max
DiBP	MiBP	421	0.95	94.3	<LOD	3.99	7.52	17.11	100.44	17138.22
DnBP	MnBP	421	0.78	100	1.62	9.70	19.30	34.70	85.69	1244.06
BBzP	MBzP	421	0.10	90.3	<LOD	0.54	1.07	2.64	12.27	162.64
DEHP	MEHP	421	0.15	99.5	<LOD	1.77	2.91	4.80	9.69	43.46
	MEOHP	421	0.05	99.7	<LOD	4.62	7.45	13.33	28.87	234.77
	MEHHP	421	0.15	100	0.47	6.27	10.52	17.91	39.27	462.52
	MECPP	394	0.12	93.6	<LOD	8.34	13.49	24.42	54.37	339.59
DiNP	MiNP	421	0.09	91.4	<LOD	0.50	0.76	1.09	1.74	11.05
	OH-MiNP	421	0.05	87.2	<LOD	0.47	0.93	1.82	5.69	74.36
	cx-MiNP	394	0.11	92.2	<LOD	0.87	1.47	2.59	7.44	219.01

a Abbreviations: LOD: Limit of detection; Max: maximum; Min: minimum; P: percentile; MiBP: monoisobutyl phthalate; MnBP: mono-*n*-butyl phthalate; MBzP: monobenzyl phthalate; MEHP: mono(2-ethylhexyl) phthalate; MEOHP: mono(2-ethyl-5-oxohexyl) phthalate; MEHHP: mono(2-ethyl-5-hydroxyhexyl) phthalate; MECPP: mono(2-ethyl-5-carboxypentyl) phthalate; MiNP: monoisononyl phthalate; OH-MiNP: monohydroxy-isononyl phthalate; cx-MiNP: monocarboxy-isononyl phthalate.

### 3.3 Individual Metabolites′ Effect: Multiple Logistic Regression Model

Individual phthalate metabolites and outcomes were assessed by multiple logistic regression, as shown in Table 3. Significant positive association was observed for allergic rhinoconjunctivitis with increased odds ratio (OR) and 95% confidence interval (95% CI) of 1.50 (1.05–2.15). While among T2 biomarkers, FeNO ≥ 35 ppb showed significant positive association with MnBP—OR and 95% CI of 1.73 (1.24– 2.45), with ∑DBP—1.41 (1.07–1.85), and with OH-MiNP—1.25 (1.04–1.52). Eosinophil count ≥ 300 cells/μL was positively associated with MnBP and all DEHP metabolites including ∑DEHP with *p* < 0.05. No significant association was observed between phthalate metabolites and ANC ≥ 4400 cells/μL.

**Table 3:** Association of Phthalate Exposure with Allergic Symptoms and T2 and Non-T2 Biomarkers

phthalate metabolites	wheeze OR (95% CI)	allergic rhinoconjunctivitis OR (95% CI)	eczema OR (95% CI)	FeNO ≥ 35 ppb OR (95% CI)	IgE ≥ 170 (IU/mL) OR (95% CI)	eosinophil ≥ 300 cells/μL OR (95% CI)	ANC ≥ 4400 cells/μL OR (95% CI)
MiBP	0.88 (0.61–1.20)	1.14 (0.93–1.38)	1.02 (0.83–1.23)	1.16 (0.97–1.40)	1.01 (0.85–1.19)	0.97 (0.82–1.16)	0.85 (0.66–1.09)
MnBP	0.83 (0.46–1.45)	1.50 (1.05–2.15)*	1.12 (0.79–1.58)	1.73 (1.24–2.45)*	1.12 (0.83–1.52)	1.49 (1.09–2.04)*	1.19 (0.79–1.78)
∑DBP	0.74 (0.43–1.19)	1.32 (0.99–1.76)	1.05 (0.78–1.38)	1.41 (1.07–1.85)*	1.00 (0.78–1.28)	1.11 (0.86–1.43 )	0.91 (0.62–1.27)
MBzP	0.98 (0.75–1.27)	1.07 (0.90–1.26)	0.95 (0.81–1.13)	1.04 (0.89–1.23)	1.07 (0.92–1.23)	0.96 (0.83–1.12)	0.88 (0.72–1.08)
MEHP	0.67 (0.36–1.22)	0.96 (0.65–1.39)	0.85 (0.58–1.24)	1.13 (0.78–1.62)	1.24 (0.90–1.72)	1.59 (1.14–2.24)*	1.00 (0.63–1.58)
MEOHP	1.24 (0.66–2.30)	1.18 (0.79–1.75)	1.14 (0.77–1.70)	1.34 (0.91–1.99)	1.21 (0.86–1.71)	1.67 (1.17–2.40)*	1.45 (0.88–2.39)
MEHHP	1.23 (0.65–2.23)	1.10 (0.73–1.64)	1.03 (0.68–1.53)	1.22 (0.82–1.80)	1.18 (0.84–1.67)	1.56 (1.10–2.24)*	1.62 (0.99–2.64)
MECPP	1.21 (0.63–2.25)	1.41 (0.93–2.13)	1.09 (0.75–1.57)	1.34 (0.88–2.01)	1.28 (0.89–1.85)	1.69 (1.16–2.48)*	1.56 (0.95–2.56)
∑DEHP	1.16 (0.58–2.20)	1.31 (0.84–2.01)	1.04 (0.68–1.60)	1.34 (0.91–1.99)	1.23 (0.86–1.75)	1.57 (1.10–2.27)*	1.54 (0.92–2.57)
MiNP	0.84 (0.57–1.25)	0.92 (0.72–1.20)	0.95 (0.74–1.23)	1.12 (0.87–1.44)	1.18 (0.95–1.47)	1.11 (0.89–1.39)	1.13 (0.82–1.57)
OH-MiNP	1.02 (0.75–1.39)	0.93 (0.77–1.13)	1.09 (0.90–1.32)	1.25 (1.04–1.52)*	1.11 (0.94–1.31)	1.09 (0.92–1.29)	1.15 (0.91–1.47)
cx-MiNP	1.08 (0.63–1.77)	0.96 (0.68–1.32)	1.14 (0.83–1.57)	1.20 (0.90–1.62)	1.10 (0.84–1.45)	1.03 (0.78–1.35)	1.14 (0.77–1.67)
∑DiNP	0.92 (0.54–1.55)	0.92 (0.65–1.27)	1.09 (0.79–1.51)	1.28 (0.95–1.74)	1.23 (0.94–1.63)	1.08 (0.82–1.43)	1.15 (0.77–1.71)

### 3.4 Mixture-Effect qgcomp and BKMR Models

**Figure 1:**
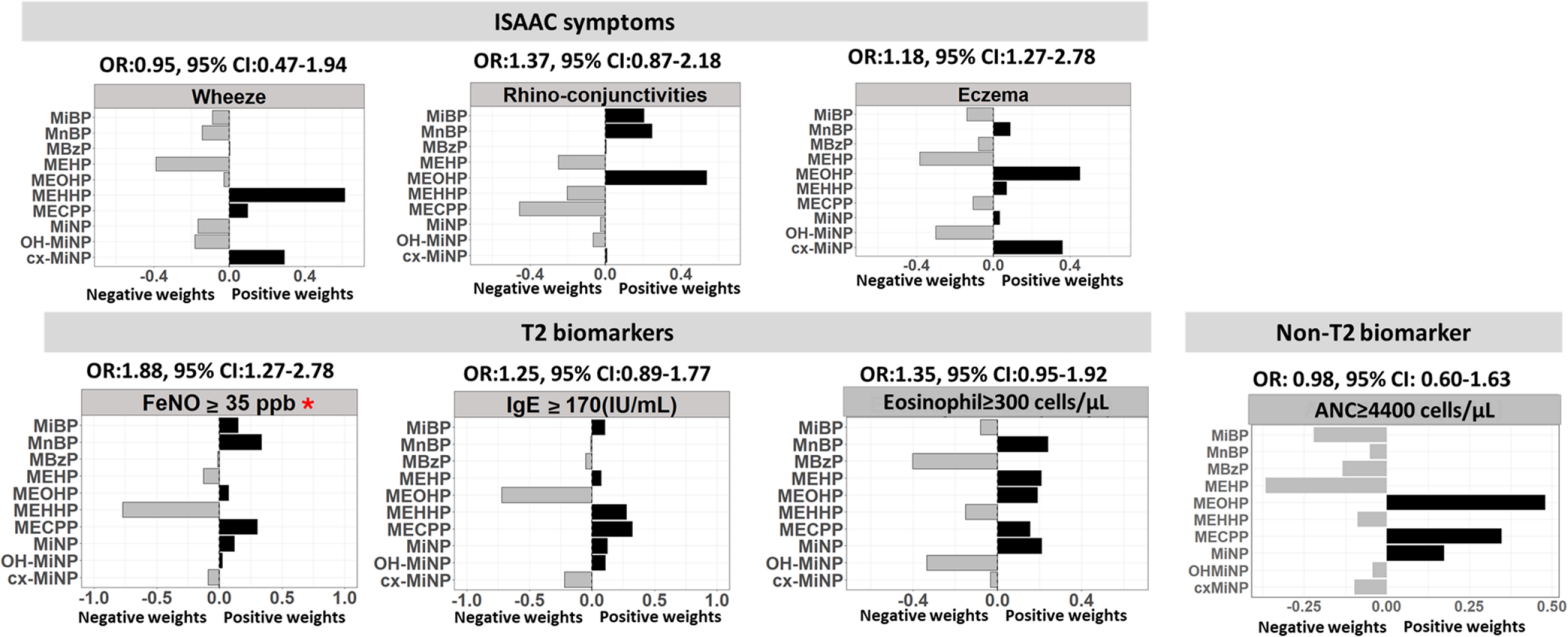
Quantile-g computation (qgcomp) mixture analysis of the relationship between the phthalate metabolite level and ISAAC symptoms. Abbreviations: ISAAC: International Study of Asthma and Allergies in Childhood; T2: type 2; OR: odds ratio; CI: confidence interval; FeNO: fraction of exhaled nitric oxide; IgE: immunoglobulin E; ANC: absolute neutrophil count; MiBP: monoisobutyl phthalate; MnBP: mono-*n*-butyl phthalate; MBzP: monobenzyl phthalate; MEHP: mono(2-ethylhexyl) phthalate; MEOHP: mono(2-ethyl-5-oxohexyl) phthalate; MEHHP: mono(2-ethyl-5-hydroxyhexyl) phthalate; MECPP: mono(2-ethyl-5-carboxypentyl) phthalate; MiNP: monoisononyl phthalate; OH-MiNP: monohydroxy-isononyl phthalate; cx-MiNP: monocarboxy-isononyl phthalate. Bars represent the contribution of each metabolite within the mixture to the outcomes. Positive contributions are indicated by darker/black bars and negative contributions are represented by gray bars. * *p* < 0.05. Models are adjusted for sex, age, BMI, ETS, and season.

**Figure 2:**
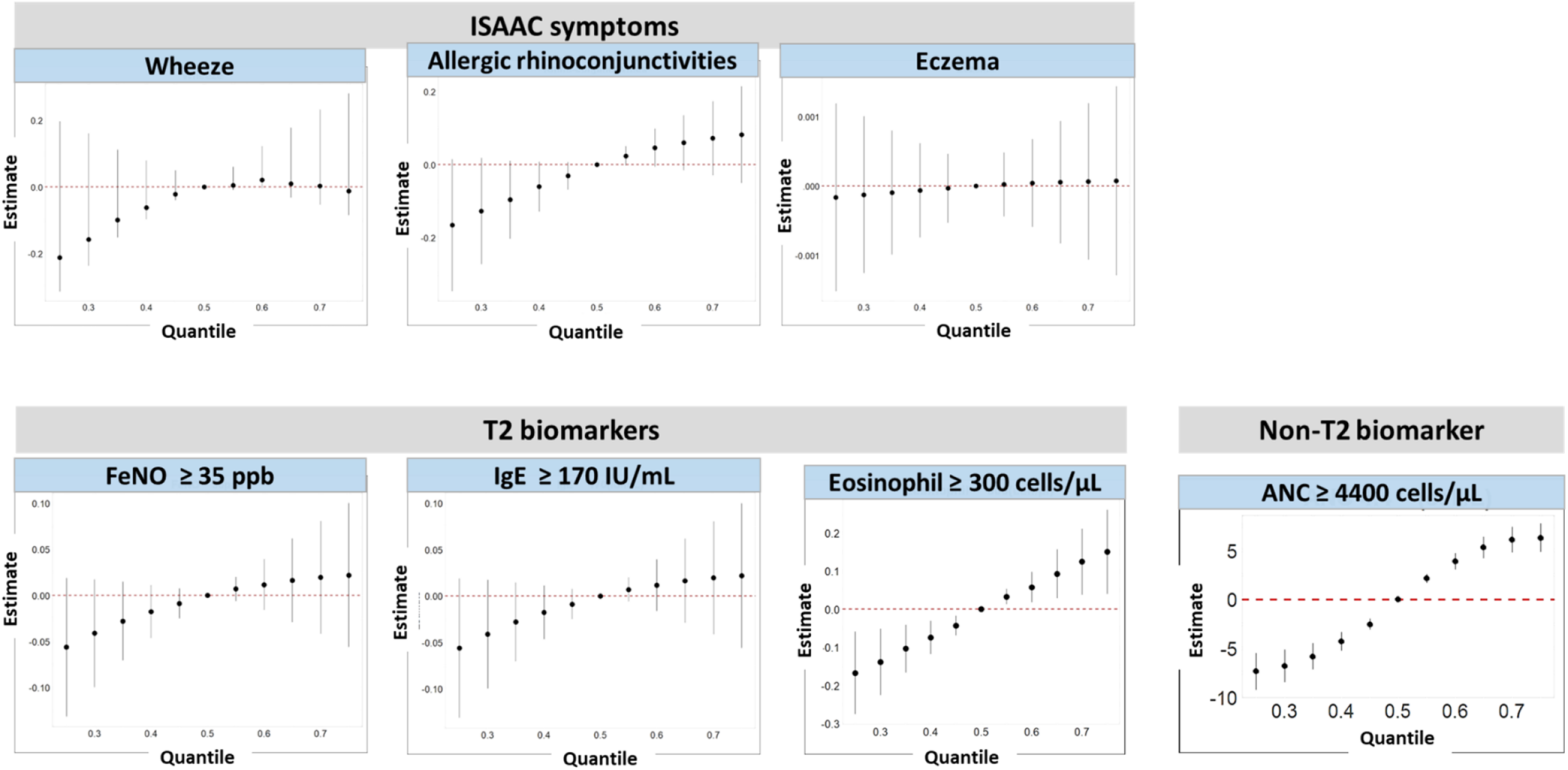
Overall mixture-effect estimate (95% CI) by Bayesian kernel machine regression (BKMR) analysis of phthalate metabolites when all of the metabolites are at particular percentile (as shown on the *x*-axis) in comparison when all metabolites were at their 50th percentile with outcomes; ISAAC symptoms, T2 and non-T2 biomarkers. Abbreviations: ISAAC: International Study of Asthma and Allergies in Childhood; T2: type 2; FeNO: fraction of exhaled nitric oxide; IgE: immunoglobulin E; ANC: absolute neutrophil count. Models are adjusted for sex, age, BMI, ETS, and season.

In the mixture-analysis qgcomp model (Figure 1), one quartile increase in the phthalate metabolite index is significantly associated with an OR of 1.88 (95% CI: 1.27–2.78) for the prevalence of FeNO ≥ 35 ppb. No significant association was observed between phthalate metabolites with other outcomes. In the mixture, the BKMR model group and individual contribution to the mixture effect are presented in Table S3. Among the mixture, DBP metabolites were the dominant group, with MnBP being the highest contributor to association with allergic rhinoconjunctivitis and FeNO ≥ 35 ppb. On the other hand, DEHP metabolites as a group were the primary contributors to association with eosinophil and ANC, with MEHP being the key driver of the association. The overall mixture model, shown in Figure 2, revealed a positive increasing trend when all metabolites were above the 55th percentile in eosinophil ≥ 300 cells/μL and ANC ≥ 4400 cells/μL compared to their 50th percentile.

## 4 Discussion

Our study found that DBP metabolite, mainly MnBP, was positively associated with an increased prevalence of allergic rhinoconjunctivitis, FeNO levels ≥ 35 ppb, and eosinophil counts ≥ 300 cells/μL, while DEHP metabolites were primarily linked to high eosinophil counts ≥300 cells/μL. Mixture analysis confirmed these findings, identifying MnBP and DEHP metabolites as key contributors to T2 and non-T2 biomarkers.

We observed a decline in phthalate metabolite concentrations in the urine samples from 9–12-year-old children in this study compared to our previous report on 7-year-old children from the same Hokkaido cohort.^29^ Considering the median levels of phthalate metabolites in urine samples collected from 9–12-year-old children (between 2017 and 2020), they decreased between 28.7 and 77.3% compared to our previous report on 7-year-old children (collected between 2012 and 2017) except MiNP, which showed a slight increase (data not shown). This decline is likely attributed to children’s age, as older children tend to have lower levels. However, this comparison between these two age groups should be interpreted cautiously; although they are participants from the same Hokkaido cohort, they are not the same individuals. Furthermore, our previous report on 7-year-old Japanese children found a stable phthalate trend^29^ and an increasing trend in phthalate alternative plasticizers from samples collected from 2012 to 2017.^36^ These findings suggest that Japan’s regulatory measures^37^ may have contributed to the observed decline of phthalate exposure in the current study, along with the market shift toward alternative plasticizers. When compared to other countries, Japanese children exhibited comparable or lower exposure levels of phthalate metabolites than those reported in Korea, China, Taiwan, the, Germany, and Australia.^38−42^ These changes indicate the need for consistent biomonitoring to track these exposure shifts over time.

Our individual regression model revealed a positive association between MnBP and allergic rhinoconjunctivitis. This was further supported by the BKMR mixture model, which identified the DBP group with MnBP as a key contributor. Epidemiological studies, including our previous report, have also demonstrated that phthalate metabolites of di-*n*-butyl phthalate (DnBP), BBzP, and DEHP increased the risk of current asthma, wheeze, eczema, and atopic dermatitis.^4−7^ These consistent positive associations between phthalates and symptoms warrant further investigation into how phthalate exposure influences asthma and allergic diseases.

We extended our analysis to examine how phthalate exposure is linked to T2 and non-T2 biomarkers concerning asthma and allergies. Notably, MnBP emerged as a key driver of T2-driven inflammation indicated in FeNO. FeNO, a marker of eosinophilic airway inflammation, is regulated by nitric oxide synthase, which is upregulated in response to T2-driven inflammation in the airways.^43^ A study in Korean children aged 10–12 year old reported a positive association between DEHP metabolites and FeNO.^44^ However, they did not find a relationship with low-molecular-weight phthalates, such as DBP metabolites. Another T2 biomarker we examined was the blood eosinophil count, which showed a positive association with MnBP and DEHP metabolites. One study reported that prenatal exposure to DEHP metabolites enhanced interleukin (IL-33) production in cord blood.^45^ The IL-33 cytokine involved in allergic diseases activates other cytokines like IL-5 and IL-13, which promote eosinophil proliferation and exacerbation of allergic inflammation.^46^ Among T2 biomarkers, the lack of association between phthalates and IgE results in our children was unexpected, given previous reports. While a similar null finding was reported in Korean children,^44^ other studies found positive associations,^15,47^ and our previous report linked maternal phthalates inversely associated with cord blood IgE.^6^ Variations in previous findings could be due to differences in demography, exposure measurement timing, or exposure levels. Nevertheless, these contradicting results highlight the need for further research on how phthalate exposure, particularly during critical development periods such as prenatal and early life, impacts allergic responses and children’s health.

Considering the non-T2 biomarker ANC, although the mixture model showed a positive trend, individual models were not significant. Notably, MBzP, a metabolite of BBzP’s was the dominant group in ANC ≥ 4400 cells/μL. In an in vitro study, BBzP-treated mice exhibited dose-dependent neutrophil extracellular trap deposition driven by reactive oxygen species increase in neutrophils, which is linked to inducing lung injury.^48^ Additionally, the NHANES data from 1999 to 2006 showed a positive association of ANC with phthalate metabolites MiBP, MnBP, MBzP, MEHHP, and MEOHP.^13^ Despite this observation, the lack of significant association between individual metabolites and ANC in this study could be due to the low phthalate metabolite concentrations, which may not trigger severe asthma typically characterized by neutrophilic phenotypes.^49^ However, it is worth mentioning that non-T2 inflammation is poorly defined^50^ and may overlap with T2 inflammation depending on the disease status.^16^ Therefore, our findings regarding neutrophils should be interpreted cautiously, as they may not fully represent the complexity of inflammatory responses.

Previous studies have suggested that phthalate exposure may induce oxidative stress, potentially affecting respiratory and allergic responses.^13,51^ Thus, we further investigated the relationship between phthalates, T2 and non-T2 biomarkers, and oxidative stress biomarkers (4-HNE, HEL, and 8-OHdG). We found significant associations between oxidative stress biomarkers and phthalates (Table S4) but no association with T2/non-T2 biomarkers (Table S5). This suggests that while phthalates may elevate oxidative stress, they do not seem to impact respiratory and immune biomarkers. However, as our study is cross-sectional, causal relationships cannot be confirmed, highlighting the need for further research into pathways that link phthalates to respiratory and allergic symptoms.

A strength of this study is its inclusion of healthy children, not limited to those with allergic diseases, which expands generalizability and offers insights into inflammatory pathways in asthma and allergies with public health implications. Additionally, utilizing diverse statistical models facilitates a broad investigation of how these exposures interact and influence outcomes, identifying dominant exposures linked to specific outcomes. Furthermore, the ISAAC questionnaires, which provide standardized data on respiratory and allergic conditions, were combined with objective T2 and non-T2 biomarkers. This approach offers a robust assessment, enhancing the reliability of our findings by incorporation of biological markers. This study also has limitations: first, the modest sample size and the focus on children aged 9–12 years may limit the generalizability of our findings to a broader population. Second, the small number of participants reporting conditions such as wheeze (31 participants, 7.5%) may limit the statistical power of our analysis, which could explain the lack of association in ISAAC outcomes in our study. However, this prevalence in our study is in line with the national survey in Japan,^52^ where the prevalence of wheeze in school-aged children is 10.2%, reflecting the general population prevalence. Third, in our study, wheeze, rhinoconjunctivitis, and eczema are defined based on questionnaires. However, the ISAAC questionnaire is a globally validated tool,^53,54^ including its application in Japanese national asthma and allergy prevalence study.^52^ Nevertheless, our objective biological measurements of FeNO, total IgE, and eosinophil and neutrophil counts enhance our findings on insights into asthma and allergies. Our findings provide valuable epidemiological evidence toward understanding the relationship of phthalates and T2 and non-T2 biomarkers with asthma and allergic symptoms. Third, BKMR and qgcomp are valuable for analyzing complex exposure interactions as they do not account for exposure concentrations, which may limit their ability to fully capture dose–response relationships. Fourth, a limitation of this study is that it did not assess other pollutants such as organophosphate flame retardants and plasticizers,^27^ polycyclic aromatic hydrocarbons,^55^ and metals and parabens,^56^ which have been associated with similar inflammatory responses.

Overall, in this study, we found that DBP and DEHP exposure are predominantly associated with allergic rhinoconjunctivitis, FeNO level, eosinophil counts, and ANC, indicating positive association with T2- and non-T2-driven respiratory and allergic responses.

## Supporting Information Available

Prevalence table on study participants’ personal and building characteristics with outcomes, distribution of oxidative stress biomarkers; BKMR model for the association of group and individual metabolites’ contributions with the outcomes; association of phthalate metabolites with oxidative stress biomarkers, and association of oxidative stress biomarkers with T2 and non-T2 inflammatory responses (PDF)
