# Supplementary material for: Association of Phthalate Exposure with Respiratory and Allergic Symptoms and Type 2 and Non-Type 2 Inflammation: The Hokkaido Study
Source: Environ Sci Technol. 2025 Apr 8;59(15):7541–9. doi: 10.1021/acs.est.4c14579 (PMC12020737; doi:10.1021/acs.est.4c14579)
Supplement: Supplementary file 1 — es4c14579_si_001.pdf [file es4c14579_si_001.pdf]

Supporting Information

Association of Phthalates Exposure with Respiratory and Allergic Symptoms, Type 2 and Non-Type 2 Inflammation: The Hokkaido Study

Rahel Mesfin Ketema [1,2], Yu Ait Bamai [2], Houman Goudarzi [3],Takeshi Yamaguchi [2], Yi Zeng [1,2,4], Ayaka Yasuda [5,6,] Megasari Marsela [1], Satoshi Konno [3], Reiko Kishi [2], Atsuko Ikeda [1,2,*]

1. Faculty of Health Sciences, Hokkaido University, Kita 12 Nishi 5, Kita-ku, Sapporo, 060-0812, Japan

2. Center for Environmental and Health Sciences, Hokkaido University, Kita 12, Nishi 7. Kita-ku, Sapporo, 060-0812, Japan

3. Faculty of Medicine, Hokkaido University, Kita 15, Nishi 7, Kita-ku, 060-8638, Sapporo, Japan

4. Creative Research Institution, Hokkaido University, North 21, West 10, Kita-ku, Sapporo, 001-0021, Japan

5. Graduate School of Pharmaceutical Sciences, Health Sciences University of Hokkaido, 1757 Kanazawa, Tobetsu-cho, Ishikari-gun, 061-0293, Japan

6. Graduate School of Health Sciences, Hokkaido University,Kita 12 Nishi 5, Kita-ku, Sapporo, 060-0812, Japan

* Corresponding author: Atsuko Ikeda, PhD

Hokkaido University, Faculty of Health Sciences

Kita 12, Nishi 5, Kita ku, Sapporo 060-0812, Japan

Phone: +81-11-706-3325

Email: AAraki@cehs.hokudai.ac.jp

Number of pages: 8 (including this page)

Number of tables: 5

Number of figures: 0

Table S1: Study population personal and building characteristics prevalence with outcomes, count (%)

Table S2: Distribution of oxidative stress biomarkers in children's urine

Table S3: Bayesian kernel machine regression (BKMR) model of group and individual contributions

Table S4: Phthalate metabolites association with oxidative stress biomarkers

Table S5: Association between T2 and non-T2 biomarkers and oxidative stress biomarkers

Table S1: Study participants' personal and building characteristics prevalence with outcomes, count (%)

Variable		Wheeze	Allergic Rhinoconjunctivities	Eczema	FeNO ≥35 ppb	IgE ≥170(IU/mL)	Eosinophil ≥300 cells/μL	ANC≥4400 cells/μL
Gender	Male	19 (8.4)	53 (23.4)	46 (20.3)	70 (30.8) *	105 (46.3)	92 (40.5)	34 (15.4)
	Female	12 (6.2)	36 (18.7)	49 (25.3)	43 (22.3)	76 (39.2)	67 (34.5)	32 (16.8)
Child age	9	12 (8.7)	27 (19.4)	32 (23.0)	35 (25.2)	62 (44.6) *	57 (41.0)	19 (14.3) *
	10	13 (7.7)	37 (22.0)	41 (24.4)	38 (22.7)	63 (37.5)	64 (38.1)	22 (13.2)
	11	4 (9.1)	12 (27.3)	11 (25.0)	18 (40.9)	27 (61.4)	18 (40.9)	6 (13.9)
	12	2 (3.0)	13 (19.1)	11 (15.7)	22 (31.4)	29 (41.4)	20 (28.6)	19 (27.1)
BMI (kg/m2)	Mean ± SD	18 ± 3.1	17.8 ± 2.7	17.6 ± 3.1	18.1 ± 3.0	17.8 ± 2.8	18.0 ± 3.2	19.3 ± 3.8 *
Annual household income (million yen)	＜3	4 (16.7)	7 (28.0)	8 (32.0)	10 (40.0)	14 (56.0)	10 (10.0)	6 (24.0)
	3-＜5	7 (7.0)	23 (23.0)	23 (22.8)	31 (30.7)	47 (46.5)	43 (42.6)	12 (12.2)
	5-＜8	14 (7.9)	34 (19.3)	45 (25.6)	50 (28.4)	75 (42.6)	71 (40.3)	29 (16.7)
	≥8	5 (5.3)	16 (16.8)	13 (13.5)	17 (17.8)	34 (35.4)	28 (29.2)	17 (18.1)
ETS	Yes	16 (9.6)	30 (17.9)	37 (21.9)	42 (24.8)	65 (38.4)	56 (33.1)	25 (14.9)
	No	15 (6.0)	59 (23.5)	58 (23.0)	71 (28.2)	116 (46.0)	103 (40.8)	41 (16.7)

Season	Winter	5 (5.8)	20 (23.0)	14 (15.9)	18 (20.5)	38 (43.2)	28 (31.8)	10 (11.5)
	Spring	3 (5.4)	14 (25.0)	11 (19.6)	13 (23.2)	24 (42.9)	20 (35.7)	10 (18.5)
	Summer	18 (9.2)	37 (19.0)	52 (26.7)	55 (28.2)	82 (42.1)	82 (42.1)	31 (16.4)
	Autumn	5 (6.2)	18 (22.2)	18 (21.9)	27 (32.9)	37 (45.1)	29 (35.4)	15 (18.3)
Pet in the house	Yes	4 (3.6) *	19 (16.9)	13 (11.6) *	29 (25.9)	39 (34.8) *	35 (31.2)	14 (12.7)
	No	27 (8.9)	70 (22.9)	82 (26.6)	83 (26.9)	141 (45.8)	123 (39.9)	52 (17.3)
PVC flooring	Yes	3 (7.9)	11 (28.9)	8 (21.1)	13 (34.2)	15 (39.5)	13 (34.2)	6 (16.7)
	No	28 (7.4)	78 (20.5)	87 (22.7)	100 (26.1)	166 (43.3)	146 (38.1)	60 (15.9)
Tatami flooring	Yes	2 (9.1)	8 (36.4)	5 (22.7)	6 (27.3)	11 (50.0)	8 (36.4)	7 (31.8) *
	No	29 (7.3)	81 (20.4)	90 (22.6)	107 (26.8)	170 (42.6)	151 (37.8)	59 (15.1)
Flooring	Yes	21 (7.3)	68 (23.4)	73 (24.9)	73 (24.9)	131 (44.7)	115 (39.3)	41 (14.3)
	No	10 (7.8)	21 (16.4)	22 (17.2)	40 (31.2)	50 (39.1)	44 (34.4)	25 (20.0)
Carpet	Yes	3 (6.2)	8 (16.7)	9 (18.8)	17 (35.4)	18 (37.5)	18 (37.5)	11 (23.9)
	No	28 (7.6)	81 (21.8)	86 (23.1)	96 (25.7)	163 (43.7)	141 (37.8)	55 (15.1)

p values calculated using chi-square test for categorical variables and Kruskal Wallice test for continuous variables

*p<0.05

Table S2: Distribution of oxidative stress biomarkers in children's urine

Oxidative stress biomarkers	Minimum	Median (IQR)	Maximum
4-HNE (µg/mL)	2.0	25.1 (13.0-41.0)	195.0
HEL (nmol/L)	4.7	100.5 (60.3-152.0)	2449.0
8-OHdG (ng/mL)	1.5	9.0 (6.9-12.7)	30.0

Abbreviations: 4-HNE: 4-hydroxynonenal, HEL: hexanoyl-lysine, 8-OHdG: 8-hydroxy-2'-deoxyguanosine, IQR: inter quartile range, levels were adjusted with creatinine

Table S3: Bayesian kernel machine regression (BKMR) model of group and individual contributions

			Wheeze		Allergic rhinoconjunctivitis		Eczema		FeNO ≥ 35ppb		IgE ≥ 170(IU/mL)		Eosinophil ≥ 300 cells/μL		ANC≥4400 cells/μL	
Parent phthalates	Metabolites	Group	Group PIP	Cond PIP	Group PIP	Cond PIP	Group PIP	Cond PIP	Grou p PIP	Cond PIP	Group PIP	Cond PIP	Group PIP	Cond PIP	Group PIP	Cond PIP
DBP	MiBP	1	0.40	0.52	0.73	0.23	0.37	0.43	0.93	0.04	0.27	0.52	0.43	0.20	0.60	0.43
	MnBP	1	0.40	0.48	0.73	0.77	0.37	0.57	0.93	0.96	0.27	0.48	0.43	0.80	0.60	0.57
BBzP	MBzP	2	0.27	1.00	0.22	1.00	0.38	1.00	0.45	1.00	0.43	1.00	0.44	1.00	1.00	1.00
DEHP	MEHP	3	0.65	0.10	0.26	0.25	0.46	0.35	0.41	0.58	0.33	0.22	0.96	0.60	0.93	0.63
	MEOHP	3	0.65	0.17	0.26	0.23	0.46	0.22	0.41	0.16	0.33	0.24	0.96	0.20	0.93	0.07
	MEHHP	3	0.65	0.53	0.26	0.16	0.46	0.25	0.41	0.14	0.33	0.29	0.96	0.10	0.93	0.12
	MECPP	3	0.65	0.21	0.26	0.36	0.46	0.18	0.41	0.12	0.33	0.25	0.96	0.09	0.93	0.17
DINP	MiNP	4	0.38	0.40	0.28	0.39	0.50	0.63	0.38	0.37	0.43	0.27	0.35	0.37	0.75	0.28
	OHMiNP	4	0.38	0.27	0.28	0.28	0.50	0.14	0.38	0.47	0.43	0.52	0.35	0.29	0.75	0.42
	cxMiNP	4	0.38	0.34	0.28	0.33	0.50	0.24	0.38	0.17	0.43	0.22	0.35	0.34	0.75	0.30

PIP: posterior inclusion probabilities for groups (group PIPs) and for individuals (condPIPs)

Table S4: Phthalate metabolites association with oxidative stress biomarkers

Oxidative stress	4-HNE		HEL		8-OHdG	
Phthalate metabolites	β	95% CI	β	95% CI	β	95% CI
MiBP	0.030	-0.026-0.086	0.004	-0.040-0.048	-0.000	-0.021-0.022
MnBP	0.157	0.052-0.262 *	0.099	0.021-0.178 *	-0.002	-0.040-0.036
∑DBP	0.081	-0.002-0.165	0.038	-0.027-0.104	-0.002	-0.034-0.029
MBzP	0.001	-0.047-0.048	0.018	-0.019-0.055	0.009	-0.008-0.027
MEHP	0.107	-0.001-0.215	0.168	0.087-0.251 *	0.052	0.023-0.092 *
MEOHP	0.160	0.043-0.277 *	0.111	0.024-0.197 *	0.061	0.020-0.103 *
MEHHP	0.209	0.092-0.326 *	0.147	0.057-0.236 *	0.064	0.021-0.107 *
MECPP	0.255	0.135-0.375 *	0.129	0.039-0.220 *	0.062	0.018-0.104 *
∑DEHP	0.240	0.115-0.365 *	0.146	0.052-0.239 *	0.063	0.019-0.108 *
MiNP	-0.068	-0.143-0.006	0.063	0.007-0.118 *	0.031	0.00-0.057 *
OH-MiNP	0.119	0.0677-0.172 *	0.002	-0.021-0.061	0.013	-0.007-0.032
cx-MiNP	0.087	-0.004-0.179	0.059	-0.008-0.128	0.044	0.012-0.077 *

∑DINP	0.097	0.006-0.189 *	0.091	0.022-0.160 *	0.042	0.009-0.075 *

Abbreviations; β: beta estimate ; CI: confidence interval; MiBP: mono-isobutyl phthalate, MnBP: mono-n-buty phthalate, MBzP: mono-benzyl phthalate, MEHP: mono (2-ethylhexyl) phthalate, MEOHP: mono (2-ethyl-5-oxohexyl) phthalate, MEHHP: mono (2-ethyl-5-hydroxyhexyl) phthalate, MECPP: mono (2-ethyl-5-carboxypentyl) phthalate, MiNP: mono-isononyl phthalate, OH-MiNP: mono-hydroxy-isononyl phthalate, cx-MiNP: mono(carboxy-isononyl phthalate),4-HNE: 4-hydroxynonenal, HEL: hexanoyl-lysine, 8-OHdG: 8-hydroxy-2'-deoxyguanosine. Adjusted for gender, age BMI, and ETS, season, *p<0.05

Table S5: Association between T2 and non-T2 biomarkers and oxidative stress biomarkers

	FeNO ≥35ppb		IgE ≥170(IU/mL)		Eosinophil ≥300 cells/µL		ANC≥4400 cells/µL	
Oxidative stress biomarkers	OR	95% CI	OR	95% CI	OR	95% CI	OR	95% CI
4-HNE	1.01	0.69-1.47	1.32	0.94-1.88	1.06	0.75-1.50	1.42	0.88-2.31
HEL	1.19	0.77-1.84	1.45	0.98-2.17	1.09	0.73-1.62	1.27	0.73-2.16
8-OHdG	0.84	0.33-2.13	1.83	0.79-4.27	0.97	0.42-2.27	1.55	0.49-4.90

Abbreviations: 4-HNE: 4-hydroxynonenal, HEL: hexanoyl-lysine, 8-OHdG: 8-hydroxy-2'-deoxyguanosine; FeNO: fraction of exhaled nitric oxide level, IgE: immunoglobulin E, ANC: absolute neutrophil count.

Adjusted for gender, age BMI, and ETS
